# p-IgGen: a paired antibody generative language model

**DOI:** 10.1093/bioinformatics/btae659

**Published:** 2024-11-09

**Authors:** Oliver M Turnbull, Dino Oglic, Rebecca Croasdale-Wood, Charlotte M Deane

**Affiliations:** Department of Statistics, University of Oxford, Oxford, OX1 3LB, United Kingdom; Centre for AI, Biopharmaceuticals R&D, AstraZeneca, Cambridge, CB2 0AA, United Kingdom; Biologics Engineering, Oncology R&D, AstraZeneca, Cambridge, CB2 0AA, United Kingdom; Department of Statistics, University of Oxford, Oxford, OX1 3LB, United Kingdom

## Abstract

**Summary:**

A key challenge in antibody drug discovery is designing novel sequences that are free from developability issues—such as aggregation, polyspecificity, poor expression, or low solubility. Here, we present p-IgGen, a protein language model for paired heavy-light chain antibody generation. The model generates diverse, antibody-like sequences with pairing properties found in natural antibodies. We also create a finetuned version of p-IgGen that biases the model to generate antibodies with 3D biophysical properties that fall within distributions seen in clinical-stage therapeutic antibodies.

**Availability and implementation:**

The model and inference code are freely available at www.github.com/oxpig/p-IgGen. Cleaned training data are deposited at doi.org/10.5281/zenodo.13880874.

## 1 Introduction

Antibodies play a crucial role in the immune response and are an increasingly important class of therapeutic (Raybould *et al.* 2024). They consist of two sets of heavy and light chains with antigen binding mediated by the Fv region of each chain [variable heavy (VH) and variable light (VL), respectively] (Chiu *et al.* 2019). The majority of the diversity in antibodies is located in six hypervariable loops within the Fv region known as complementarity determining regions (CDRs). The light chain and heavy chain each contain three CDR loops (CDRL 1–3 and CDRH 1–3).

Modern antibody drug discovery typically relies on large libraries of paired VH and VL sequences, which are screened for affinity against a target (Zhang 2023). However, such libraries often contain sequences with developability issues (Jain *et al.* 2017) (i.e. propensity for aggregation, polyspecificity, poor expression, or low solubility), which can result in potential therapeutics being discarded or requiring engineering later in the pipeline (Raybould *et al.* 2024). These developability properties depend on the combination of the heavy and light chain.

Protein language models (PLMs) have proven effective in a variety of tasks. Masked PLMs are trained with the task of predicting masked-out tokens (in the case of PLMs, residues) in a sequence and thereby learn a rich representation of protein sequences. Masked PLMs have proven effective for tasks such as property prediction (Meier *et al.* 2021) and suggesting evolutionarily plausible mutations (Hie *et al.* 2023). Auto-regressive (AR) models are trained to predict the next residue in the sequence and during inference are able to generate full sequences. AR PLMs have demonstrated the ability to generate novel and diverse protein sequences (Ferruz *et al.* 2022, Nijkamp *et al.* 2023, Shuai *et al.* 2023).

Here, we present paired-IgGen (p-IgGen), an AR PLM trained on both unpaired and paired antibody sequences. p-IgGen generates diverse and antibody-like paired sequences, as measured with a variety of sequence and structure-based metrics. Our extensive validation indicates the paired sequences generated by p-IgGen follow similar pairing patterns to those seen in natural paired sequences. To make best use of available antibody sequence data, we use a pretraining regime capable of ingesting the large corpus of unpaired sequences (∼250M) followed by finetuning on the smaller but more biologically relevant paired sequence data (∼1.8M). The model can also be biased to generate sequences with desired properties through finetuning. Here, we bias the model to generate antibodies with 3D biophysical properties that fall within distributions seen in clinical-stage therapeutic antibodies, as predicted by the structure-based developability predictor the Therapeutic Antibody Profiler (TAP) (Raybould *et al.* 2019), which we release as ‘developable p-IgGen’. Finally, we show that p-IgGen outperforms other antibody language models on zero-shot prediction benchmarks, demonstrating robust sequence representations.

## 2 Materials and methods

### 2.1 Model and training

We trained three models: IgGen, p-IgGen, and developable p-IgGen. IgGen was pretrained on unpaired sequences and finetuned on paired sequences to give p-IgGen. p-IgGen was further finetuned on a set of developable sequences to give developable p-IgGen. All models use the same autoregressive decoder-only architecture based on GPT-2 (Brown *et al.* 2020) with the addition of rotary positional embeddings (Su *et al.* 2024) which have been shown to improve PLM performance (Hayes *et al.* 2024, Olsen *et al.* 2024), implemented in PyTorch. We used three attention layers, each with 12 attention heads and an embedding size of 768, the feed-forward layers had a dimension of 2048, for a total of 17 349 888 parameters. Sequences were tokenized at the residue level, with a special token added to the start (‘1’) and end (‘2’) of each sequence. For the paired models, the light chain was concatenated to the heavy chain. During training, we randomly provided each sequence in either the forward or reverse direction. By training in both directions, a heavy chain can be generated given a light chain and vice versa during inference.

All training was performed using the Adam optimizer with a cosine learning rate scheduler. IgGen was trained for 20 epochs on 4 A100 GPUs with a learning rate of 1E-4, a local batch size of 512 and 4 gradient accumulation steps. p-IgGen was trained by finetuning all layers of IgGen for three epochs using a batch size of 256 and a learning rate of 1E-5 on an A100 GPU. Finally, developable p-IgGen was trained by finetuning all layers of p-IgGen for two epochs with the same hyperparameters as used to train p-IgGen. We selected the minimum number of fine-tuning epochs that provided the desired distributional changes while minimizing catastrophic forgetting of the pretraining sequences. For p-IgGen, this was determined by evaluating the true vs. randomly paired validation sequence likelihood and for developable p-IgGen by observing a shift in the TAP distribution of generated sequences.

### 2.2 Model sampling

We found that using a sampling temperature of 1.2, a top-p of 0.95, and then discarding the bottom 5% of generated sequences according to the model likelihood best represented natural antibody sequences in terms of mutation rate and diversity, while remaining valid. For developable p-IgGen, we increased the sampling temperature to 1.25 to maintain diversity. We used 2000 sequences for all comparisons.

### 2.3 Data

Models were trained using antibody sequences taken from the Observed Antibody Space (OAS) (Olsen *et al.* 2022). Only human sequences were used, and sequences were filtered to reduce redundancy and to remove sequences which likely contained PCR sequencing errors (see Supplementary Information for full filtering details). For the unpaired dataset, this resulted in 117 431 915 VL and 130 246 252 VH sequences. The filtered paired dataset consists of 1 800 545 VH/VL sequences.

For the developable dataset, we structurally modelled all sequences from paired OAS using ABodyBuilder2 (Abanades *et al.* 2023). The structures were then flagged for developability using TAP (Raybould *et al.* 2019), which calculates five metrics which have been associated with poor developability. CDR sequences that had green flags for the four structure-based metrics (PSH, SFvCSP, PPC, and PNC) were classified as developable and included in the finetuning set. We did not filter on the CDR length metric, as this is sequence-based so generated sequences could be quickly and easily filtered for this. As p-IgGen had already been trained on the paired dataset, we ensured that we kept the same train/validation/test splits as used for training the paired model.

Datasets for zero-shot prediction tasks were taken from the FLAb repository (Chungyoun *et al.* 2024). The immunogenicity set consists of the anti-drug antibody response against 217 therapeutics curated by Marks *et al.* (2021). The expression dataset is taken from Koenig *et al.* (2017) and consists of a deep mutational scan of an anti-vascular endothelial growth factor (VEGF) antibody, with 4275 sequences. There was no overlap between the paired zero-shot test sequences and the unpaired or paired training set sequences.

## 3 Results

### 3.1 Paired antibody language model

p-IgGen is an AR decoder-only language model using a GPT-2 like architecture (Brown *et al.* 2020), see Section 2 for full architecture and training details. We trained p-IgGen in a two-step procedure, first pretraining on the much larger available dataset of unpaired sequences. ‘IgGen’ was trained on a filtered set of 117M VL and 140M VH sequences taken from the OAS (Olsen *et al.* 2022), see Section 2 for full filtering and tokenization details. ‘p-IgGen’ was then trained by finetuning IgGen on a set of 1.8M paired VH/VL sequences taken from OAS.

### 3.2 p-IgGen generates novel, realistic, and diverse paired sequences

We evaluated the sequences generated by p-IgGen using a comprehensive set of *in silico* metrics, demonstrating that its generated sequences are unique, diverse, and antibody-like. By comparing these metrics against a test set of natural paired sequences, we establish that the distributions of generated sequences are very similar to those of natural sequences.

We found that sequences generated by p-IgGen were as similar to natural sequences as natural sequences are to each other, as measured by Hamming distance (Table 1). Generated sequences also show a similar sequence identity to both training and validation sets, indicating that the model has not overfit to the training data (see Supplementary Figs S4 and S5). Following Shin *et al.* (2021), we examined the diversity of generated sequences using the cosine similarity with each sequence’s nearest neighbour. At a sampling temperature of 1.2, the generated sequences were as diverse as natural sequences (see Table 1, Supplementary Fig. S9). We found that generated sequences had a slight skew to higher novelty for the VL relative to natural sequences (Supplementary Fig. S5). This diversity can also be tuned by adjusting the sampling temperature.

**Table 1.** Comparison of metrics between p-IgGen sequences, randomly paired p-IgGen sequences, and natural sequences.^a^

Metric	p-IgGen sequences	Randomly paired p-IgGen sequences	Natural sequences
Mean VH Hamming distance to val set	11.3_*n.s.*_	11.3_*n.s.*_	11.3
Mean VL Hamming distance to val set	10.3_*n.s.*_	10.3_*n.s.*_	9.5
Diversity	0.16_*n.s.*_	0.17*	0.16
ESM-2 likelihood	−0.33_*n.s.*_	−0.33_*n.s.*_	−0.33
VH-VL mutation rate correlation	0.52	−0.04	0.51

a We compared a variety of metrics investigating the novelty, diversity, and validity of sequences. As a control, we randomly paired the VH and VL chains from the ‘p-IgGen Sequences’ set. Natural sequences were taken from the training set. Diversity is measured by the mean cosine distance between sequences. Mann–Whitney *U* tests were performed to check for significant differences between natural and p-IgGen generated sequence metrics, with * indicating a significant difference (*P* < 0.05) and *n.s.* indicating non-significance. VH-VL mutation rate correlation was calculated by looking at the Pearson correlation coefficient between the number of mutations away from germline in the VH and VL chains and was not subject to the Mann–Whitney *U* test.

Generated sequences show a similar distribution of ESM-2 (Lin *et al.* 2023) likelihoods, suggesting that they are just as ‘protein-like’ as natural sequences (Supplementary Fig. S7). To assess if they were antibody-like, we aligned and numbered all sequences with the antibody-numbering tool ANARCI (Dunbar and Deane 2015), which successfully identified a heavy and light chain for all sequences. The distribution of CDR lengths was also examined and found to be closely aligned with natural sequences (Supplementary Fig. S6). We also tested whether the sequences could be structurally modelled using ABodyBuilder2 (ABB2) (Abanades *et al.* 2023) and found similar confidence values as those seen for natural sequences (Supplementary Fig. S8).

Finally, we investigated whether the generated sequences show VH/VL pairing characteristics similar to those observed in natural sequences. Naturally paired sequences show a correlation in the mutation rates of the heavy and light chains, relative to their respective germline sequences. We found that generated sequences from p-IgGen also display a similar correlation, while no such correlation is observed when the same generated sequences are randomly paired together (see Table 1, Supplementary Fig. S10). To further evaluate p-IgGen’s ability to recognize true VH-VL pairings, we compared the model’s likelihood scores for true pairings against those of 50 randomly selected VL sequences for each VH, controlling for mutation rates (see Supplementary Material for full details). In 94% of cases, the true VH-VL pairing had a higher likelihood than the mean of randomly paired sequences. For 12% of VH sequences, the true VL pairing had the highest likelihood out of the 51 pairings, and in 52% of cases, it was within the top 8. These results indicate that p-IgGen captures VH/VL pairing preferences beyond mutation rate matching. This suggests that sequences generated by p-IgGen are not only antibody-like but also have biologically plausible VH/VL pairings.

The validation described above of p-IgGen’s generated sequences spans a broad array of tests: assessing sequence identity and diversity, protein-likeness, antibody-specific properties (including ANARCI numbering and CDR length distributions), and conducting structural modelling with ABB2. Together, these tests confirm that p-IgGen generates novel and diverse sequences that appear just as antibody-like as natural sequences. This comprehensive validation confirms p-IgGen’s potential to generate novel, realistic, and diverse paired antibody libraries.

### 3.3 Generation can be biased towards antibodies with desired sequence and structure-based properties

Having verified that p-IgGen can create diverse, realistic, and previously unseen antibodies, we then investigated whether the generation space could be restricted to antibodies with desirable developability properties. The approach we took was to fine-tune p-IgGen on a set of antibodies with the desired properties. This has the advantage of being very simple to implement; it does not require a differentiable property predictor or reinforcement learning. As a case study, we fine-tuned on a set of developable antibodies, as predicted by the TAP tool (Raybould *et al.* 2019). Specifically, we structurally modelled all 1.8M paired sequences using ABB2 and ran these structures through TAP. We defined an antibody as ‘developable’ if it had all green flags for the four structure-based TAP metrics (PSH, PPC, PNC, and SFvCSP) (see Section 2 for full details). We used this ‘safe’ set of 909 790 sequences to fine-tune paired p-IgGen to create a ‘developable p-IgGen’. The developable p-IgGen is therefore trained in a three-step process—first pretrained on unpaired data, then finetuned on paired sequences, and finally finetuned on highly developable sequences.

**Figure 1.**
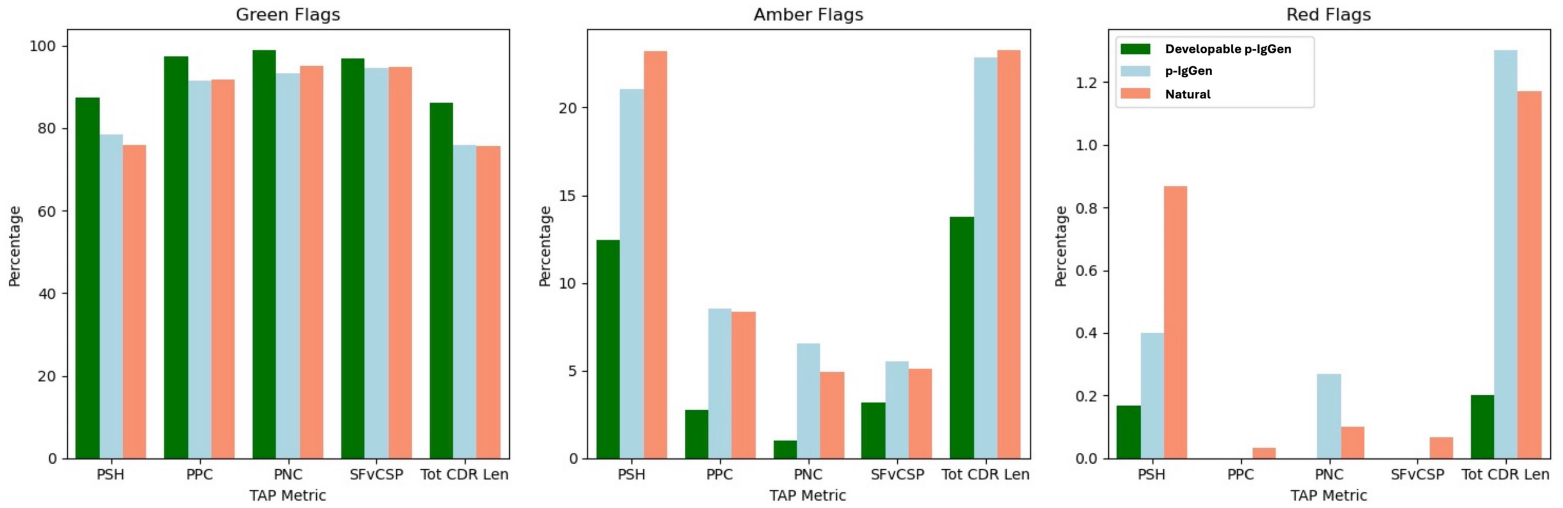
Developable p-IgGen shows favourable TAP flagging compared to p-IgGen on both structure-based and sequence-based metrics. We generated 3000 sequences from p-IgGen, and developable p-IgGen and sampled 3000 sequences from the paired OAS validation set. All of these sequences were structurally modelled with ABB2 and were then run through TAP to generate developability flags for the four structure-based metrics (PSH, PPC, PNC, SFvCSP) and the total CDR length. Green flagged sequences represent low developability risk, amber flagged are medium risk, and red flagged are high risk.

Finetuning on the developable set shifted the distribution of the 3D biophysical properties of generated antibodies despite being trained on sequence alone. We saw a significant reduction in the proportion of amber and red-flagged antibodies for all metrics for the developable model relative to the paired model (Fig. 1). The diversity of generated antibodies was still maintained, as measured by intraset diversity and sequence identity (see Supplementary Figs S11 and S13).

### 3.4 p-IgGen achieves state-of-the-art performance on zero-shot tasks

Finally, to understand whether our models were learning meaningful representations of antibodies, we tested their ‘zero-shot’ prediction performance on two antibody fitness datasets. We used a deep mutational scan dataset of 4275 anti-VEGF antibodies (Koenig *et al.* 2017) to assess zero-shot prediction of expression, and a curated dataset of antidrug antibody responses for 217 therapeutic antibodies for immunogenicity prediction (Marks *et al.* 2021). For zero-shot accuracy, we looked at the Pearson correlation of the perplexity of sequences under the given model with the fitness metric being assessed.

For model testing and comparison, we used the Fitness Landscapes for Antibodies (FLAb) testing suite (Chungyoun *et al.* 2024). FLAb offers benchmark results for various state-of-the-art models, both sequence-based [IgLM (Shuai *et al.* 2023), AntiBERTy (Ruffolo *et al.* 2021), ProGen (Nijkamp *et al.* 2023)] and structure-based [ProteinMPNN (Dauparas *et al.* 2022), ESM-IF (Hsu *et al.* 2022)]. However, FLAb lacks benchmarks for structure-based models on the expression dataset. For both datasets, we found that p-IgGen outperformed IgGen, while p-IgGen and developable p-IgGen performed similarly. For the expression dataset, p-IgGen outperformed all other antibody-specific LMs (AntiBerty, IgLM, and ProGen OAS) (see Supplementary Information). The ProGen general protein LMs outperformed p-IgGen for expression prediction, but even the smallest ProGen model has more than 7.5× the number of parameters of p-IgGen and requires significantly more compute to train (see Supplementary Information for full comparison). The superior performance of general PLMs for expression suggests the evolutionary patterns learnt during training on diverse proteins may be important for the more general problem of protein expression. For the immunogenicity dataset, p-IgGen outperformed all other methods, with the same performance for both the developable p-IgGen model and the p-IgGen model (Table 2). This improved immunogenicity prediction may be due to two factors: the models’ exclusive training on human antibody sequences, thereby scoring non-human immunogenic sequences as more unlikely, and training on paired sequences. It has been hypothesized that paired sequences offer a more accurate representation of human-like antibodies than unpaired data (Chinery *et al.* 2024). Results for all FLAb prediction categories can be found in Supplementary Fig. S14.

**Table 2.** p-IgGen and developable p-IgGen outperform all other models for zero-shot prediction of immunogenicity.^a^

Model	Parameters	Pearson correlation
p-IgGen	17M	0.53
Developable p-IgGen	17M	0.52
ProGen/small	151M	0.48
ProGen/medium	764M	0.46
IgGen	17M	0.45
ProGen/xlarge	6.4B	0.34
ProGen/oas	764M	0.29
ESM-IF	124M	0.28
IgLM	13M	0.20
ProGen/large	2.7B	0.07
ProGen/base	764M	0.06
MPNN	1.7M	−0.03
AntiBerty	26M	−0.05

a Language models and inverse folding models (ESM-IF and MPNN) were evaluated for zero-shot prediction of immunogenicity using a dataset of antidrug antibody responses for 217 therapeutic antibodies curated by Marks *et al*. (2021) using FLAb. Results are ordered by Pearson’s correlation (best to worst).

## 4 Conclusions

In this work, we have presented and extensively validated p-IgGen, an antibody language model capable of producing realistic paired sequences and achieving state-of-the-art performance on zero-shot tasks. The ability to finetune p-IgGen to produce sequences with desired biophysical properties while preserving diversity highlights its applicability to high throughput antibody drug discovery.

## Supplementary Material

btae659_Supplementary_Data

## Data Availability

The data underlying this article are available in the Observed Antibody Space (OAS), at https://opig.stats.ox.ac.uk/webapps/oas. The filtered and cleaned data for training and testing have been deposited on Zenodo https://doi.org/10.5281/zenodo.13880874.
