# Supplementary material for: p-IgGen: a paired antibody generative language model
Source: Bioinformatics. 2024 Nov 9;40(11):btae659. doi: 10.1093/bioinformatics/btae659 (PMC11576349; doi:10.1093/bioinformatics/btae659)
Supplement: btae659_Supplementary_Data [file btae659_supplementary_data.pdf]

Appendix

1 Tokenisation Scheme

Forward Tokenisation	Reverse Tokenisation
1{VH}2	2{Reverse VH}1
1{VL}2	2{Reverse VL}2
1{VH}{VL}2	2{Reverse VL}{Reverse VH}1

Table 1: **Tokenisation Scheme** IgGen is provided with VH and VL sequences separately, while p-IgGen and developable p-IgGen are provided with the VL concatenated to the VH. All sequences are provided in the forward direction as well as reversed. During training, models are shown all sequences in both the forward and reverse direction.

2 Dataset Filtering

For unpaired OAS, heavy and light sequences were filtered separately to remove identical sequences and any sequences marked by ANARCI [Dunbar and Deane, 2015] as having shorter than IMGT defined framework region 1 or 4, missing conserved cysteines, or containing unknown residues. The sequences were then further filtered for redundancy by clustering at 95% identity using linclust [Steinegger and Söding, 2018] with coverage mode 1 (target coverage). Within each cluster, we further clustered by identical CDRs and kept a random sample for each sub-cluster. We numbered sequences with the IMGT scheme using ANARCI [Dunbar and Deane, 2015] and used IMGT CDR definitions [Lefranc et al., 2003]. 117,431,915 VL and 130,246,252 VH sequences were used for further steps.

Paired OAS was filtered to remove sequences with missing conserved cysteine residues or with unknown residues. Sequences with deletions in framework regions were completed using AbLang [Olsen et al., 2022b]. This was not performed for unpaired sequences as a large amount of data was already available. Due to the smaller size of paired OAS, and the increased diversity relative to unpaired OAS due to the combination of both VH and VL chains for each sequence, we did not filter the sequences for redundancy, apart from ensuring no identical full VH/VL sequences were present. For the train, validation, and

test splits, we clustered length matched CDRs at 95% sequence identity using cd-hit [Li and Godzik, 2006].

3 Computational Requirements

Pretraining on unpaired sequences took approximately 4 days using 4 A100s. As a comparison, Progen-OAS required a $\sim$ 45-fold higher training budget. Fine-tuning on paired sequences took only four hours on a single A100. Using 8 CPU cores, 10,000 sequences can be generated in 21 minutes. With an A100 GPU, 10,000 sequences can be generated in approximately 3.5 minutes. Generating a library larger than all of paired OAS (2 million sequences) would therefore take less than 12 hours using an A100.

4 Temperature Sensitivity

We investigated a variety of sampling temperatures and found that a temperature of 1.2 gave the most 'natural' like generated sequences while maintaining novelty (Figure 1).

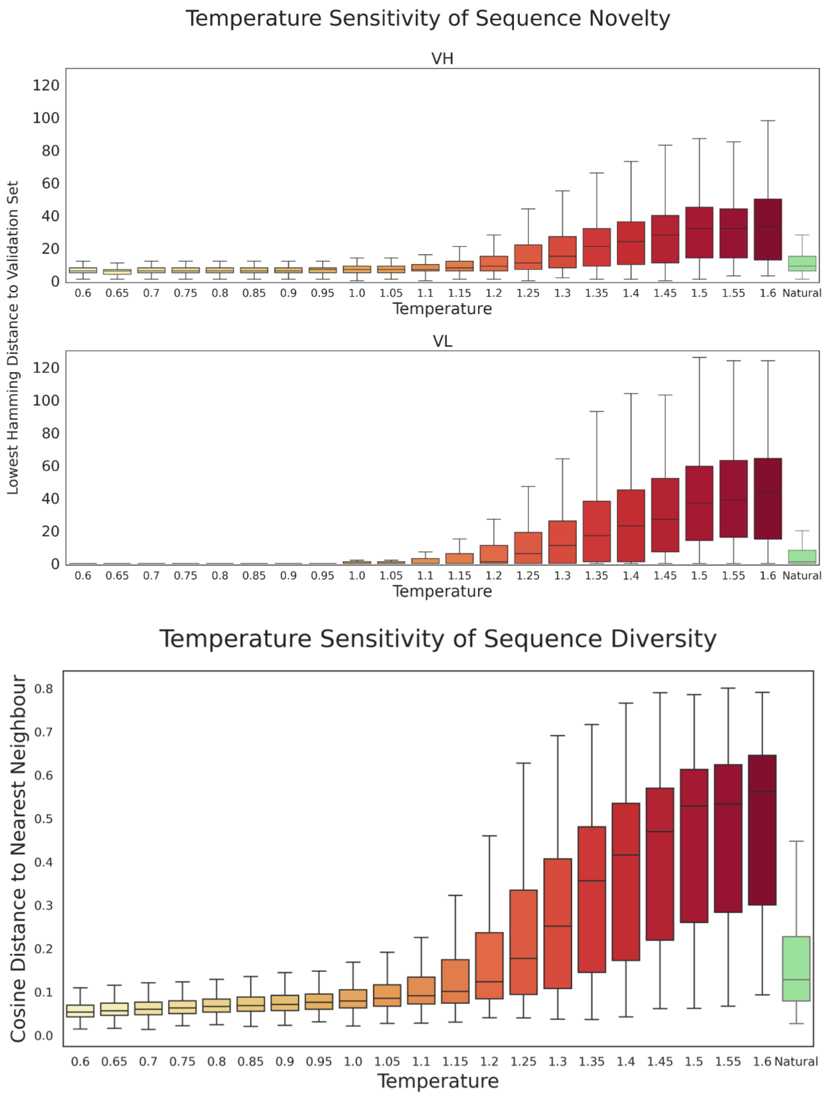

Figure 1: **Sensitivity of sequence diversity and novelty to sampling temperature.** We generated 2000 sequences from p-IgGen at sampling temperatures ranging from 0.60 to 1.40 at 0.05 increments. Novelty was defined as the lowest Hamming distance of generated VH and VL chains to the training set. 'Natural' novelty was calculated by randomly sampling 2000 test set sequences and calculating the minimum Hamming distance to train set sequences. Diversity was measured by the lowest pairwise cosine distance within generated or sampled test set sequences ('natural').

5 Sequence Validation

We generated samples using top-p sampling, as implemented in the HuggingFace Transformers library [Wolf et al., 2020], with a top-p value of 0.95, and a temperature value of 1.2 unless otherwise stated. We then calculated the model likelihood of generated sequences and discarded the bottom 5% of sequences. We numbered sequences using ANARCI [Dunbar and Deane, 2015] and the IMGT scheme [Lefranc et al., 2003] to identify the heavy and light chains and allow for other downstream analyses.

Sequence novelty was measured by the lowest Hamming distance of generated VH and VL chains to sequences in the train and validation set. We compared each generated VH and VL chain to all length-matched chains in the train and validation set and took the lowest value.

To assess the intraset diversity of the generated and test sequences, we calculated the pairwise cosine diversity of 3-mer subsequences of the paired sequences within each set, with the light chain concatenated after the heavy chain, using the scikit-learn library [Pedregosa et al., 2011]. We calculated the pseudo-log-likelihood of sequences using ESM2 [Lin et al., 2023], with the light chain concatenated after the heavy chain using the esm2_t12_35M_UR50D model hosted on HuggingFace [Wolf et al., 2020].

We used ANARCI-derived numbering and IMGT definitions to calculate the length distribution of the CDRs within the generated and natural sets. ANARCI annotations were also used for germline gene usage and sequence identity to germline sequences. We modelled all generated sequences using ABodyBuilder2 (ABB2) [Abanades et al., 2023] and extracted error estimates from the generated pdb files. To predict developability we ran the generated structures through the Therapeutic Antibody Profiler (TAP) [Raybould et al., 2019].

5.1 Pairing Validity

To investigate whether p-IgGen has learnt biologically relevant pairing properties, we looked at (1) the correlation between VH and VL mutation rates in generated sequences, and (2) the model's ability to distinguish true VH-VL pairings from random ones through likelihood.

5.1.1 VH-VL Mutation Rate Calculation

We calculated the VH-VL mutation rate by determining the number of mutations away from the germline sequence using ANARCI [Dunbar and Deane, 2015]. We found a similar correlation for both natural and p-IgGen sequences and no correlation for randomly paired generated sequences (Figure 10).

5.1.2 True vs. Random Pairing Likelihood

To evaluate p-IgGen's ability to recognise true VH-VL pairings, we compared the model's likelihood scores for true parings against those of random pairings.

As we have already shown p-IgGen has learnt to match mutation rates across the VH and VL chain, we controlled for this by matching the mutation rates of the randomly selected VL chains to those of the true VL chains.

We first randomly selected a set of 4,000 paired sequences from the OAS test set and then binned these sequences based on the VL V-gene germline identity ('100-95%', '95-90%', '90-80%', '<80%'). We randomly paired each VH seqeunce with 50 VL sequences from the same VL v-gene identity bin as the true VL. We then calculated the probability of each VH given either true or random VL pairings ($p(VH|VL)$).

For 94% of sequences, the true VH-VL pairing $p(VH_i|VL_{true})$ was higher than the mean $p(VH_i|VL_{random})$ of randomly paired sequences. As a control, we compared the $p(VL)$ of the true vs randomly selected VL chains for each VH. We found that only 66% of true VLs had a higher likelihood than the mean of the randomly selected VL for a given VH. We also looked at the $p(VH|VL_{true})$ ranking for each VH. For 12% of sequences, $p(VH|VL_{true})$ was ranked as the most likely pairing, and for 52% of VHs it was ranked within the top 8 (out of 51) pairings. We would not expect a perfect ranking here, as the test set will likely contain false negatives, randomly paired VH-VL chains that would in fact make a good pairing.

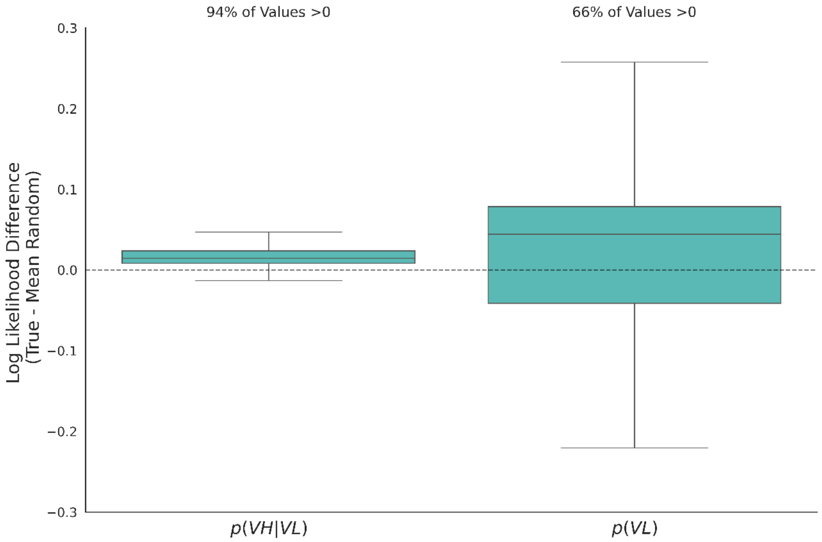

Figure 2: **Log likelihood difference between true and random pairings.** We looked at the difference in likelihood between the true VH-VL and the mean of the random pairings for each VH. The left boxplot shows $p(VH|VL)$ where 94% of values are above 0, indicating that true pairings are more likely than the mean of random ones. The right boxplot shows the difference in p(VL) for the true VL compared to randomly chosen VLs for each VH, where only 66% of values are above 0. This indicates the model is not relying on the true VL being more likely independent of the VH chain.

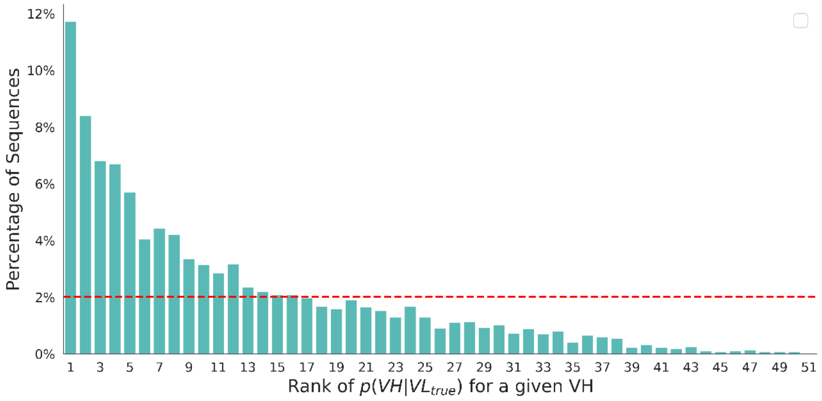

Figure 3: **Ranking distribution of $p(VH|VL_{true})$ for each VH.** The x-axis shows the rank of the true VL pairing among 50 random pairings, while the y-axis shows the percentage of sequences. The red dashed line indicates the expected percentage (2%) if rankings were uniformly distributed.

VH-VL mutation rate correlation was calculated by looking at the Pearson correlation coefficient between the number of mutations away from germline in the VH and VL chains and was not subject to the Mann-Whitney U test.

5.2 Statistical Testing

We performed Mann-Whitney U tests to check for significant differences between the distribution of properties of generated sequences and natural sequences.

Metric	Generated vs. Natural	Generated (random pairing) vs. Natural
Diversity	0.354	$\mathbf{2.00 \times 10^{-10}}$
VH Sequence Identity	0.511	0.511
VL Sequence Identity	0.467	0.466
ESM-2 Likelihood	0.728	0.678

Table 2: Mann-Whitney U Test Results Comparing Natural and Generated Sequences. P-values are shown for each metric, with significant results (p <0.05) shown in bold.

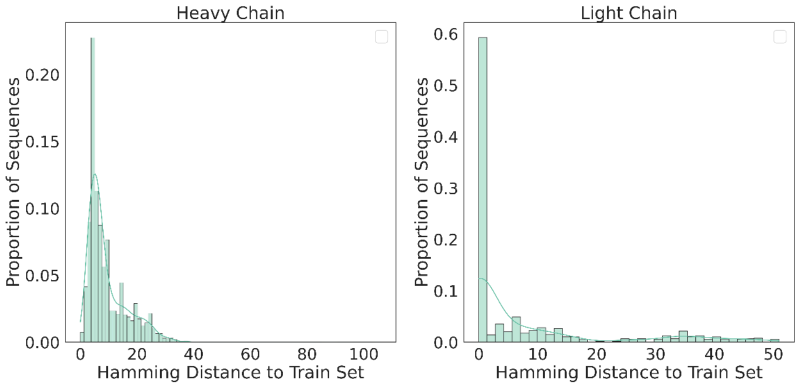

Figure 4: **Generated sequences do not show signs of overfitting to the training sequences.** We calculated the minimum Hamming distance (i.e. closest sequence) of VH and VL chains from 2,000 sequences generated by p-IgGen with the paired OAS training set. VH and VL regions were extracted from the generated sequences using ANARCI. KDE lines show the smoothed distribution of the sequence identity data.

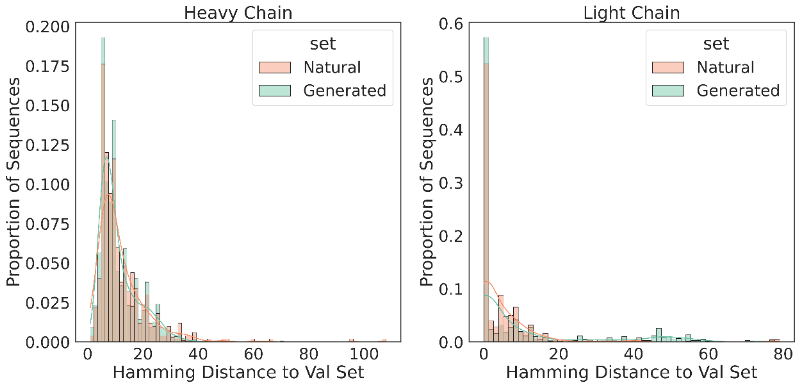

Figure 5: **Generated sequences show similar distances to validation set sequence as training set sequences do to validation set sequences.** We calculated the minimum Hamming distance (i.e. closest sequence) of VH and VL from 2,000 sequences generated by p-IgGen with the paired OAS validation set (”Generated”). We also calculated the sequence identity of a random sample of 2,000 OAS paired training set sequences to validation set sequences (”Natural”). VH and VL regions were extracted from the generated sequences using ANARCI. KDE lines show the smoothed distribution of the sequence identity data.

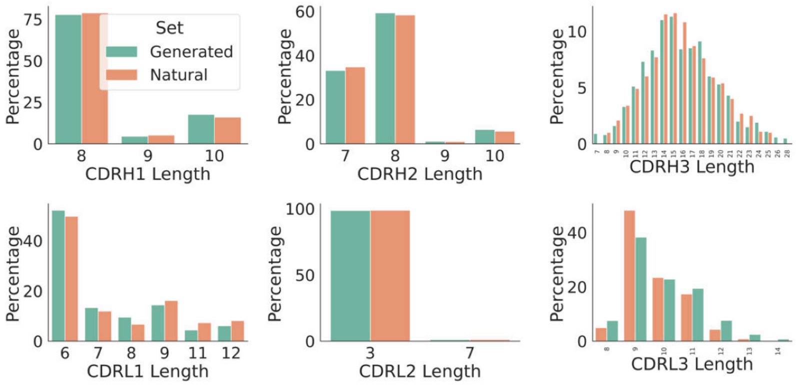

Figure 6: **Generated sequences show a similar distribution of CDR lengths to natural sequences.** We looked at the distribution of CDR lengths of sequences generated by p-IgGen (“Generated”) compared to test set sequences from paired OAS (“Natural”). Lengths were determined using IMGT-defined CDR positions with IMGT numbering using ANARCI.

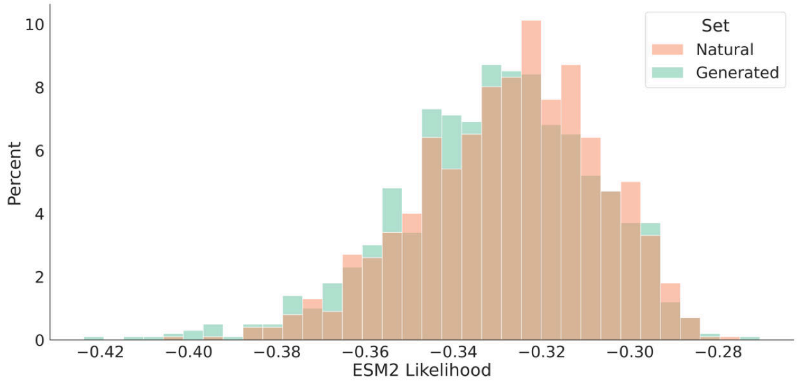

Figure 7: **Generated sequences have a similar distribution of ESM-2 log-likelihoods as natural sequences.** We calculated the log-likelihood of 2,000 full VH/VL sequences generated by p-IgGen ("Generated") as well as 2,000 sequences taken from the test set of paired OAS using the masked protein language model ESM-2.

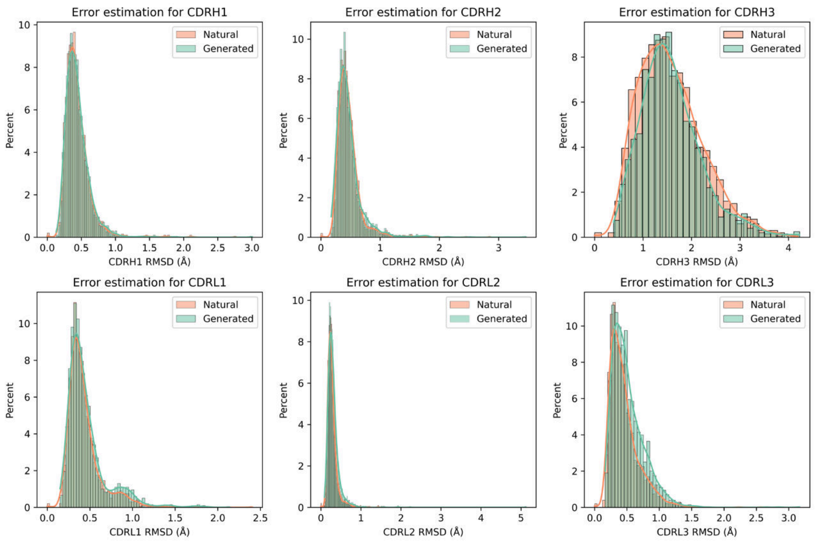

Figure 8: **Generated sequences have similar structural modelling error estimates as natural sequences.** We structurally modelled 2,000 generated and 2,000 natural sequences using ABB2. Per loop error estimates were produced by taking the mean ABB2 RMSD error estimate across residues in IMGT-defined CDR regions, as numbered by ANARCI.

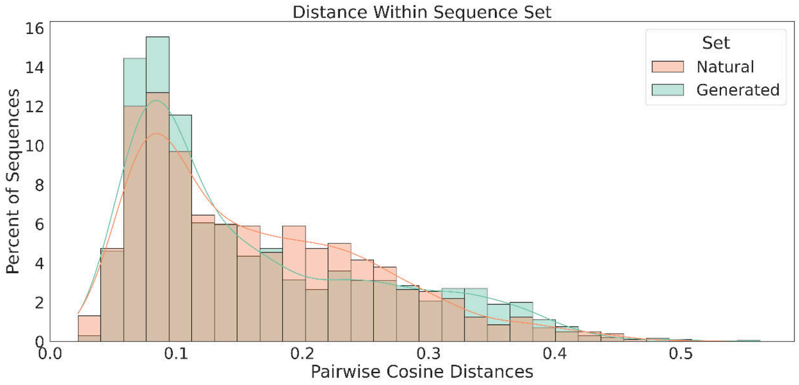

Figure 9: **Intraset diversity, measured by cosine distance, is similar for generated and natural sequences.** We calculated the highest pairwise cosine distance for 2,000 sequences generated from p-IgGen using a sampling temperature of 1.2 (“Generated”). This was compared to the highest pairwise cosine distance for 2,000 sequences sampled from the validation set of paired OAS (“Natural”). KDE lines show the smoothed distribution of the diversity data.

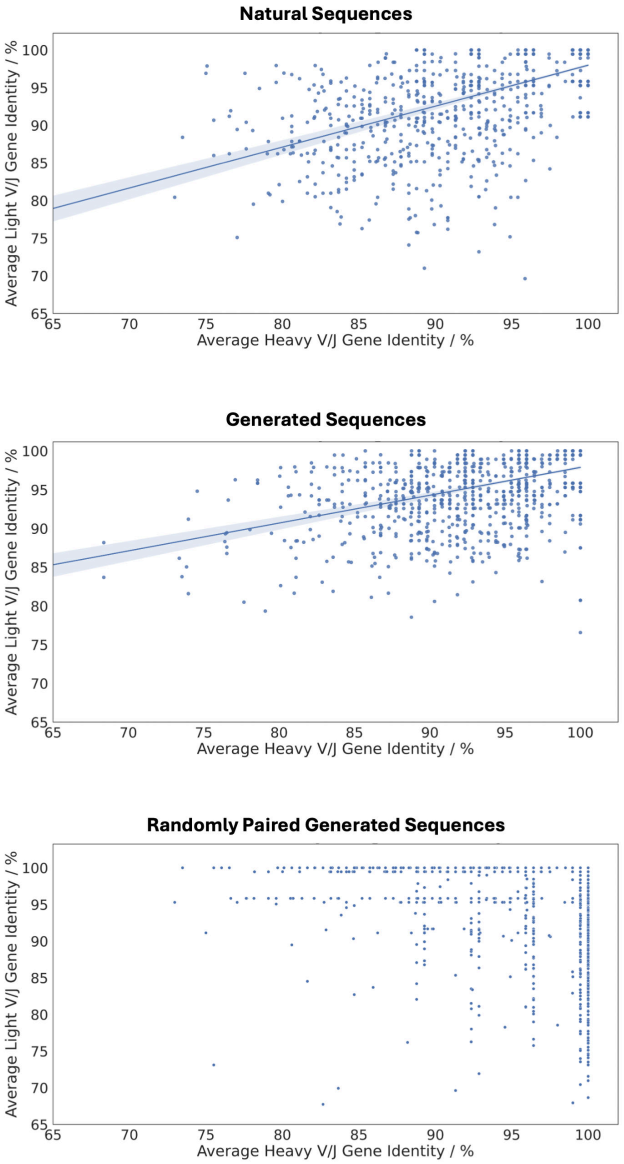

Figure 10: **Both natural sequences and paired sequences generated by p-IgGen show a correlation between the mutation rates of the VH and VL chains.** Average V/J gene identity to germline was used as a measure of mutation of the VH and VL chains, as reported by ANARCI. Natural sequences (taken from the paired OAS validation set) and sequences generated by p-IgGen ("Generated Sequences") show a strong correlation between the VH and VL mutation rates. No correlation is seen for generated sequences with randomly paired VH and VL chains.

6 Property Biasing

We found that after fine-tuning, a slightly higher sampling temperature of 1.25 was needed to achieve diversity similar to natural sequences. However, a lower diversity is also expected as we've restricted the generation space to developable antibodies.

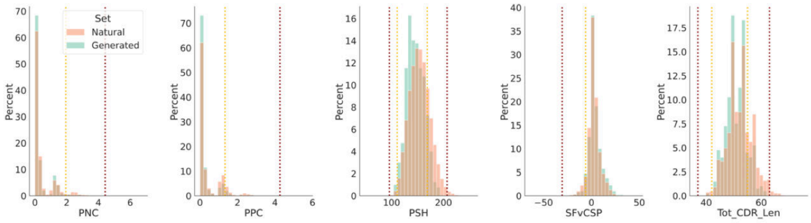

Figure 11: **Antibodies generated by developable p-IgGen show a favourable shift in the distribution of TAP metrics relative to natural sequences.** We generated and structurally modelled 2,000 sequences from developable p-IgGen using ABB2. We then ran TAP on the structural models to calculate the four structure-based metrics (PNC, PPC, PSH, and SFvCSP) and the total CDR length ("Generated"). We also calculated the TAP metrics for all paired OAS test set sequences ("Natural") using the same methodology.

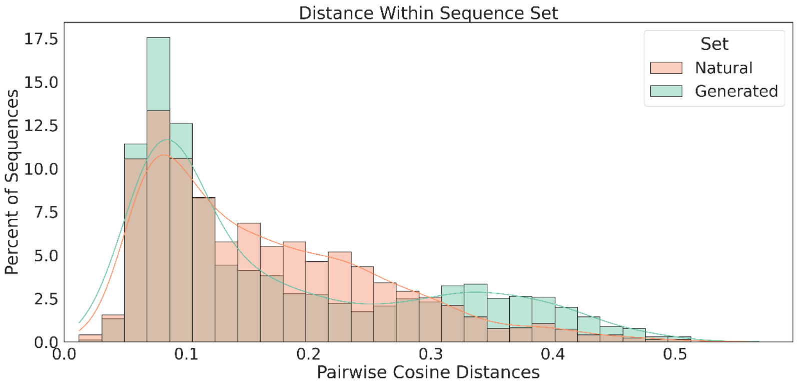

Figure 12: **Sequences generated by developable p-IgGen maintain their diversity.** We calculated the highest pairwise cosine distance for 2,000 sequences generated from developable p-IgGen using a sampling temperature of 1.25 ("Generated"). This was compared to the highest pairwise cosine distance for 2,000 sequences sampled from the validation set of developable paired OAS ("Natural").

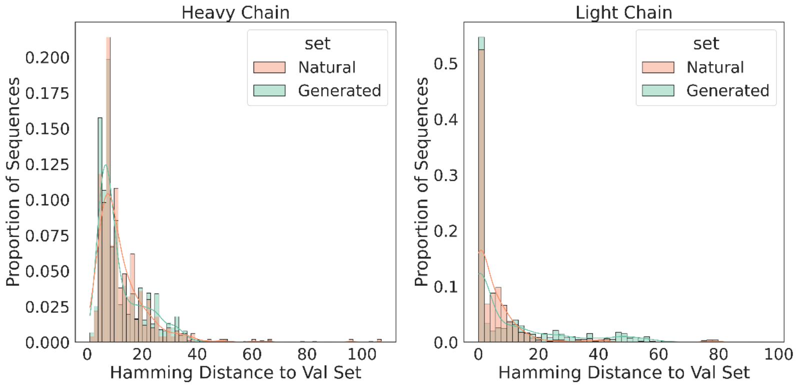

Figure 13: **Sequences generated by developable p-IgGen maintain a similar Hamming distance distribution as seen with p-IgGen.** We calculated the minimum Hamming distance (i.e. closest sequence) of VH and VL chains from 2,000 sequences generated by developable p-IgGen with the validation set of developable paired OAS. We also calculated the sequence identity of a random sample of 2,000 developable paired OAS training set sequences to validation set sequences ("Natural").

7 Zero-shot Task

For zero-shot prediction, we adapted code from the FLAb repository [Chungyoun et al., 2024] to calculate the perplexity of paired sequences in each dataset and calculated the Pearson's correlation coefficients (PCCs) with the experimental assay data. For IgGen we calculated the mean perplexity of the VH and VL sequences. For the p-IgGen models, we took the perplexity of the concatenated VH and VL sequences. We focused on the Koenig et al. expression dataset due to its size (n=4275) and the immunogenicity dataset. Antibody expression, assessed using ELISA fluorescent signal, measures the efficiency of antibody production in host cells, which directly impacts manufacturing yield and cost. Immunogenicity, quantified as the percentage of patients developing anti-drug antibodies (ADA) in response to therapeutic administration, indicates the likelihood of an immune response to the antibody treatment, with an ideal, non-immunogenic antibody resulting in no ADA response.

We also present the results from all six prediction categories of FLAb (binding, Tm, expression, immunogenicity, polyreactivity, aggregation) in Figure 14. We took the absolute value of the PCC and omitted any correlations which were not statistically significant ($p<0.05$). Note, that the immunogenicity category consists of only one dataset, and for aggregation only ESM-IF1 and ProGen/xlarge had statistically significant correlations.

Model	Parameters	Pearson Correlation
ProGen/small	151M	0.56
ProGen/medium	764M	0.56
ProGen/base	764M	0.53
ProGen/xlarge	6.4B	0.50
ProGen/large	2.7B	0.49
Developable p-IgGen	17M	0.42
p-IgGen	17M	0.41
IgGen	17M	0.28
AntiBerty	26M	0.27
IgLM	13M	0.27
ProGen/oas	764M	0.20

Table 3: **The p-IgGen models (p-IgGen and developable p-IgGen) significantly outperform the unpaired IgGen model and other state-of-the-art language models of comparable size for zero-shot expression prediction.** Language models were evaluated for zero-shot prediction of expression levels with a deep mutational scan dataset consisting of 4275 anti-VEGF antibodies [Koenig et al., 2017] using FLAb. Results are ordered by Pearson's correlation (best to worst).

**Model**	**Parameters**	**Training Dataset(s)**
AntiBerty	26M	Unpaired OAS
IgGen	17M	Unpaired OAS
p-IgGen	17M	Unpaired OAS, Paired OAS (finetuning)
developable p-IgGen	17M	Unpaired OAS, Paired OAS (finetuning)
IgLM	13M	Unpaired OAS
ProGen/oas	764M	Unpaired OAS
ProGen/small	151M	UniRef90, BFD30
ProGen/medium	764M	UniRef90, BFD30
ProGen/base	764M	UniRef90, BFD30
ProGen/large	2.7B	UniRef90, BFD30
ProGen/xlarge	6.4B	UniRef90, BFD30
ESM-IF	124M	CATH40, UniRef50
MPNN	1.7M	PDB

Table 4: **Summary of model parameters and training data.** Inverse folding models (ESM-IF and MPNN) were trained on structural data, while all other models were trained on sequence data. AntiBerty, IgLM, and Progen-OAS were trained on unpaired antibody sequences from OAS [Olsen et al., 2022a]. All other ProGen models were trained on UniRef90 [Suzek et al., 2015], a redundancy-filtered subset of the UniProt dataset, and BFD30, which is mainly from metagenomic sources [Steinegger and Söding, 2018]. ESM-IF was trained on experimental structures from CATH40 [Sillitoe et al., 2015], a redundancy-filtered subset of the Protein Data Bank (PDB) [Berman et al., 2000], as well as AlphaFold2 [Jumper et al., 2021] predicted structures of UniRef40 [Suzek et al., 2015]. MPNN was trained on a subset of experimental structures taken from the PDB.

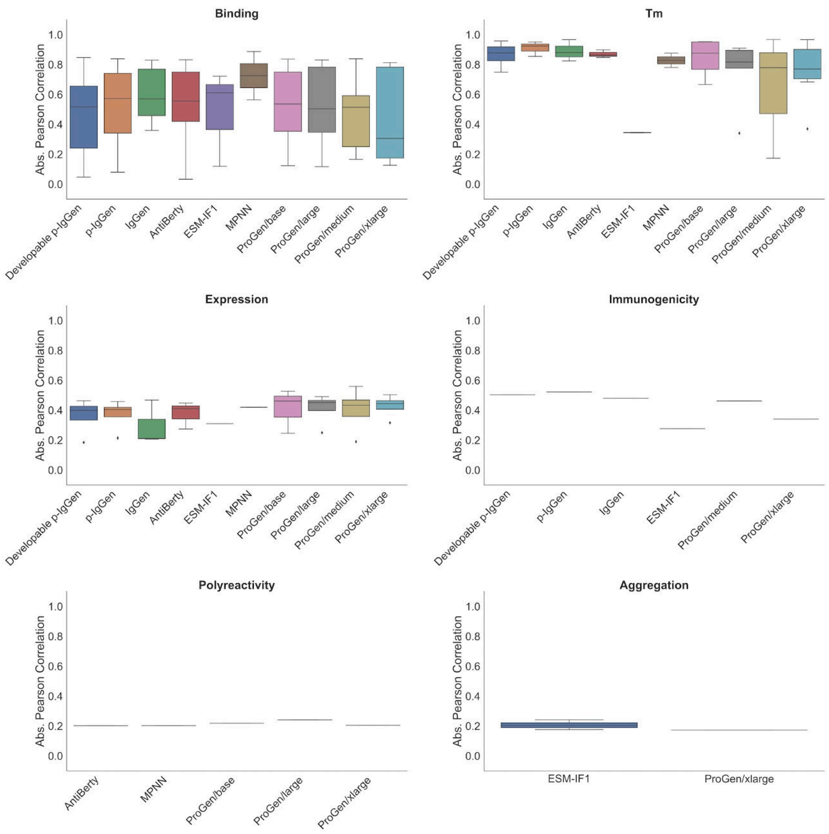

Figure 14: **Pearson correlation across all FLAb benchmark sets.** Only Pearson correlation coefficients which are statistically significant (p<0.05) are displayed.

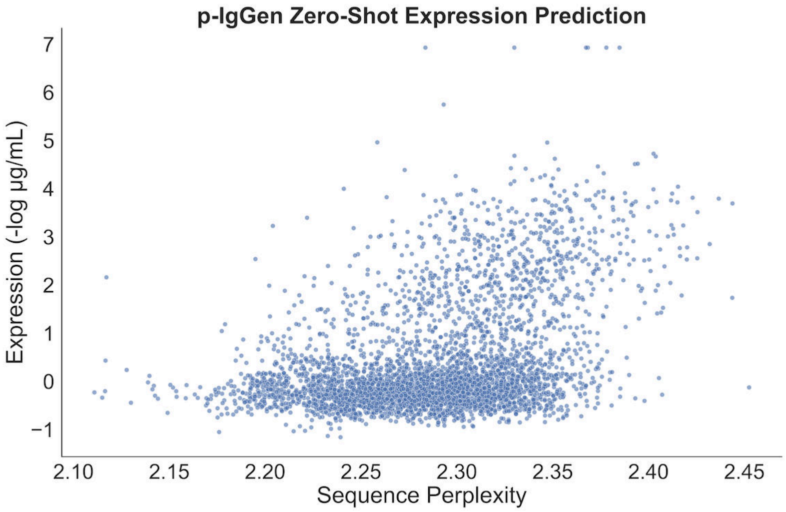

Figure 15: **p-IgGen sequence perplexity against expression.**

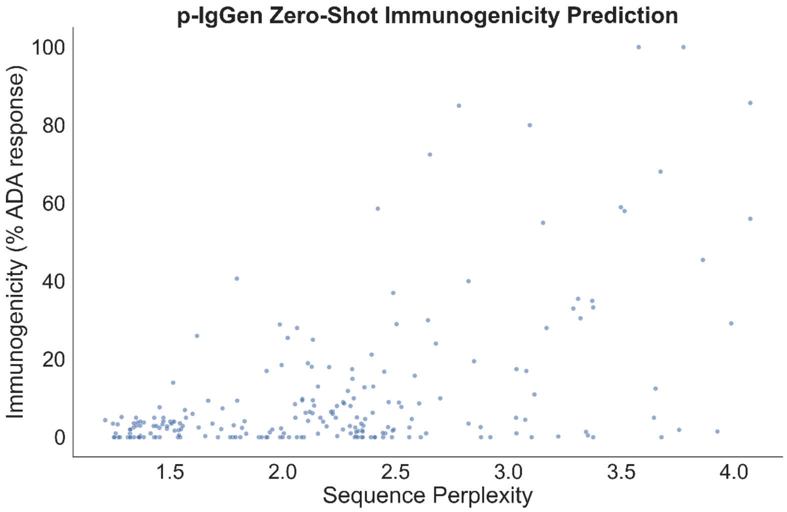

Figure 16: **p-IgGen sequence perplexity against immunogenicity.**

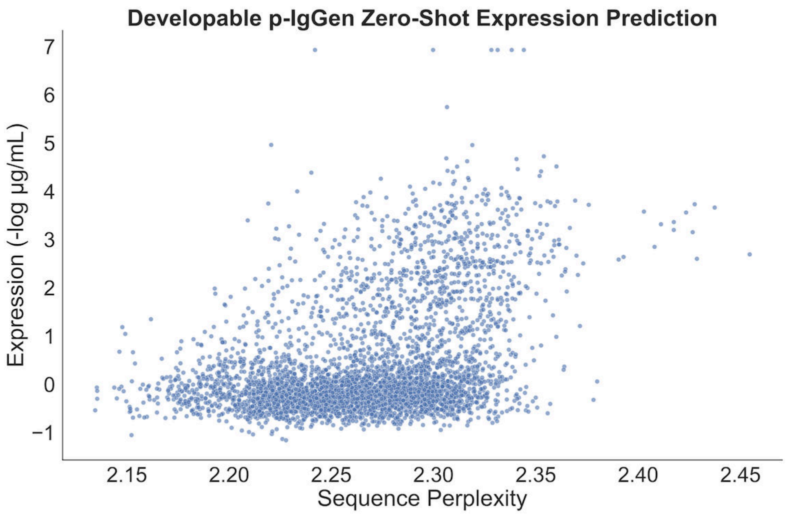

Figure 17: **Developable p-IgGen sequence perplexity against expression.**

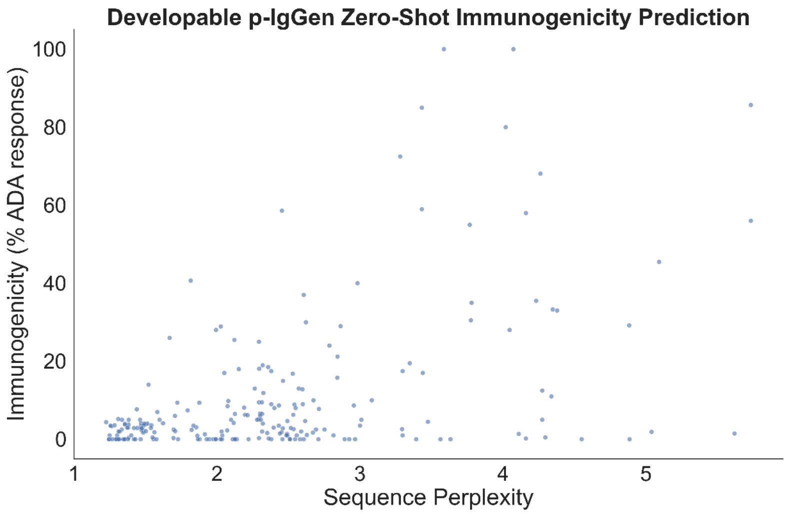

Figure 18: **Developable p-IgGen sequence perplexity against immunogenicity.**

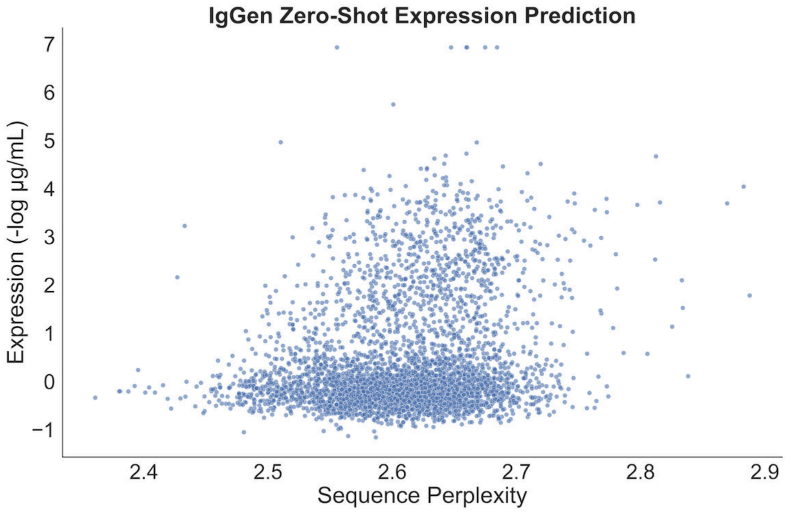

Figure 19: **IgGen sequence perplexity against expression.**

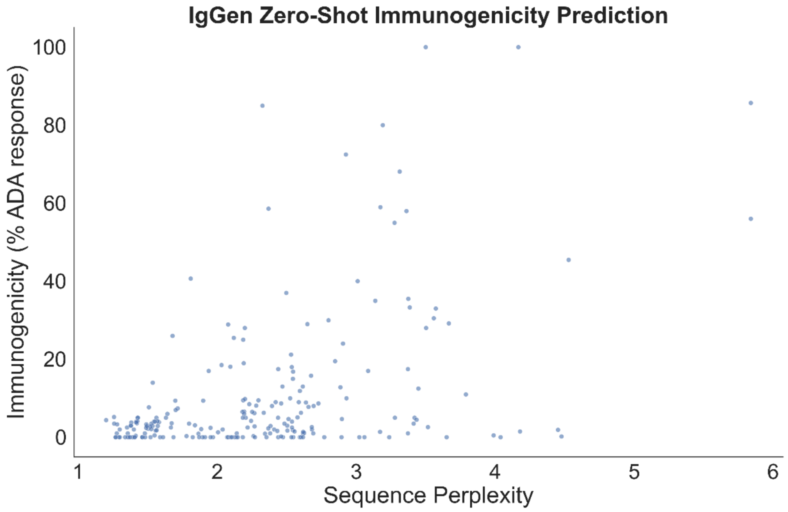

Figure 20: **IgGen sequence perplexity against immunogenicity.**

References

B. Abanades, W. K. Wong, F. Boyles, G. Georges, A. Bujotzek, and C. M. Deane. ImmuneBuilder: Deep-Learning models for predicting the structures of immune proteins. *Communications Biology*, 6(1):1–8, May 2023. ISSN 2399-3642. doi: 10.1038/s42003-023-04927-7. URL https://www.nature.com/articles/s42003-023-04927-7. Number: 1 Publisher: Nature Publishing Group.

H. M. Berman, J. Westbrook, Z. Feng, G. Gilliland, T. N. Bhat, H. Weissig, I. N. Shindyalov, and P. E. Bourne. The Protein Data Bank. *Nucleic Acids Research*, 28(1):235–242, Jan. 2000. ISSN 0305-1048. doi: 10.1093/nar/28.1.235. URL https://doi.org/10.1093/nar/28.1.235.

M. Chungyoun, J. Ruffolo, and J. Gray. FLAb: Benchmarking deep learning methods for antibody fitness prediction, Jan. 2024. URL https://www.biorxiv.org/content/10.1101/2024.01.13.575504v1. Pages: 2024.01.13.575504 Section: New Results.

J. Dunbar and C. M. Deane. ANARCI: antigen receptor numbering and receptor classification. *Bioinformatics*, page btv552, Sept. 2015. ISSN 1367-4803, 1460-2059. doi: 10.1093/bioinformatics/btv552. URL https://academic.oup.com/bioinformatics/article-lookup/doi/10.1093/bioinformatics/btv552.

J. Jumper, R. Evans, A. Pritzel, T. Green, M. Figurnov, O. Ronneberger, K. Tunyasuvunakool, R. Bates, A. Žídek, A. Potapenko, A. Bridgland, C. Meyer, S. A. A. Kohl, A. J. Ballard, A. Cowie, B. Romera-Paredes, S. Nikolov, R. Jain, J. Adler, T. Back, S. Petersen, D. Reiman, E. Clancy, M. Zielinski, M. Steinegger, M. Pacholska, T. Berghammer, S. Bodenstein, D. Silver, O. Vinyals, A. W. Senior, K. Kavukcuoglu, P. Kohli, and D. Hassabis. Highly accurate protein structure prediction with AlphaFold. *Nature*, 596(7873):583–589, Aug. 2021. ISSN 1476-4687. doi: 10.1038/s41586-021-03819-2. URL https://www.nature.com/articles/s41586-021-03819-2. Number: 7873 Publisher: Nature Publishing Group.

P. Koenig, C. V. Lee, B. T. Walters, V. Janakiraman, J. Stinson, T. W. Patapoff, and G. Fuh. Mutational landscape of antibody variable domains reveals a switch modulating the interdomain conformational dynamics and antigen binding. *Proceedings of the National Academy of Sciences of the United States of America*, 114(4):E486–E495, Jan. 2017. ISSN 1091-6490. doi: 10.1073/pnas.1613231114.

M.-P. Lefranc, C. Pommié, M. Ruiz, V. Giudicelli, E. Foulquier, L. Truong, V. Thouvenin-Contet, and G. Lefranc. IMGT unique numbering for immunoglobulin and T cell receptor variable domains and Ig superfamily V-like domains. *Developmental & Comparative Immunology*, 27(1):55–77, Jan. 2003. ISSN 0145305X. doi: 10.1016/S0145-305X(02)00039-3. URL https://linkinghub.elsevier.com/retrieve/pii/S0145305X02000393.

W. Li and A. Godzik. Cd-hit: a fast program for clustering and comparing large sets of protein or nucleotide sequences. *Bioinformatics (Oxford, England)*, 22 (13):1658–1659, July 2006. ISSN 1367-4803. doi: 10.1093/bioinformatics/btl158.

Z. Lin, H. Akin, R. Rao, B. Hie, Z. Zhu, W. Lu, N. Smetanin, R. Verkuil, O. Kabeli, Y. Shmueli, A. dos Santos Costa, M. Fazel-Zarandi, T. Sercu, S. Candido, and A. Rives. Evolutionary-scale prediction of atomic-level protein structure with a language model. *Science*, 379(6637):1123–1130, Mar. 2023. doi: 10.1126/science.ade2574. URL `https://www.science.org/doi/10.1126/science.ade2574`. Publisher: American Association for the Advancement of Science.

T. H. Olsen, F. Boyles, and C. M. Deane. Observed Antibody Space: A diverse database of cleaned, annotated, and translated unpaired and paired antibody sequences. *Protein Science*, 31(1):141–146, 2022a. ISSN 1469-896X. doi: 10.1002/pro.4205. URL `https://onlinelibrary.wiley.com/doi/abs/10.1002/pro.4205`. _eprint: https://onlinelibrary.wiley.com/doi/pdf/10.1002/pro.4205.

T. H. Olsen, I. H. Moal, and C. M. Deane. AbLang: an antibody language model for completing antibody sequences. *Bioinformatics Advances*, 2(1): vbac046, Jan. 2022b. ISSN 2635-0041. doi: 10.1093/bioadv/vbac046. URL `https://doi.org/10.1093/bioadv/vbac046`.

F. Pedregosa, G. Varoquaux, A. Gramfort, V. Michel, B. Thirion, O. Grisel, M. Blondel, P. Prettenhofer, R. Weiss, V. Dubourg, J. Vanderplas, A. Passos, D. Cournapeau, M. Brucher, M. Perrot, and E. Duchesnay. Scikit-learn: Machine learning in Python. *Journal of Machine Learning Research*, 12: 2825–2830, 2011.

M. I. J. Raybould, C. Marks, K. Krawczyk, B. Taddese, J. Nowak, A. P. Lewis, A. Bujotzek, J. Shi, and C. M. Deane. Five computational developability guidelines for therapeutic antibody profiling. *Proceedings of the National Academy of Sciences*, 116(10):4025–4030, Mar. 2019. doi: 10.1073/pnas.1810576116. URL `https://www.pnas.org/doi/10.1073/pnas.1810576116`. Publisher: Proceedings of the National Academy of Sciences.

I. Sillitoe, T. E. Lewis, A. Cuff, S. Das, P. Ashford, N. L. Dawson, N. Furnham, R. A. Laskowski, D. Lee, J. G. Lees, S. Lehtinen, R. A. Studer, J. Thornton, and C. A. Orengo. CATH: comprehensive structural and functional annotations for genome sequences. *Nucleic Acids Research*, 43(Database issue): D376–381, Jan. 2015. ISSN 1362-4962. doi: 10.1093/nar/gku947.

M. Steinegger and J. Söding. Clustering huge protein sequence sets in linear time. *Nature Communications*, 9(1):2542, June 2018. ISSN 2041-1723. doi: 10.1038/s41467-018-04964-5.

B. E. Suzek, Y. Wang, H. Huang, P. B. McGarvey, C. H. Wu, and the UniProt Consortium. UniRef clusters: a comprehensive and scalable alternative for improving sequence similarity searches. *Bioinformatics*, 31(6):926–932, Mar. 2015. ISSN 1367-4803. doi: 10.1093/bioinformatics/btu739. URL `https://doi.org/10.1093/bioinformatics/btu739`.

T. Wolf, L. Debut, V. Sanh, J. Chaumond, C. Delangue, A. Moi, P. Cistac, T. Rault, R. Louf, M. Funtowicz, J. Davison, S. Shleifer, P. von Platen, C. Ma, Y. Jernite, J. Plu, C. Xu, T. L. Scao, S. Gugger, M. Drame, Q. Lhoest, and A. M. Rush. HuggingFace's Transformers: State-of-the-art Natural Language Processing, July 2020. URL `http://arxiv.org/abs/1910.03771`. arXiv:1910.03771 [cs].
